# Synergistic Lethality Effects of Apatinib and Homoharringtonine in Acute Myeloid Leukemia

**DOI:** 10.1155/2022/9005804

**Published:** 2022-08-30

**Authors:** Yuanfei Shi, Dandan Xu, Yi Xu, Huafei Shen, Yan Zhang, Xiujin Ye, Jie Jin, Dawei Cui, Wanzhuo Xie

**Affiliations:** ^1^Department of Hematology, The First Affiliated Hospital, College of Medicine, Zhejiang University, Hangzhou, Zhejiang, China; ^2^Department of Blood Transfusion, The First Affiliated Hospital, College of Medicine, Zhejiang University, Hangzhou, Zhejiang, China

## Abstract

**Purpose:**

The significance of vascular endothelial growth factor receptor (VEGFR)-2 in numerous solid tumors and acute myeloid leukemia (AML) has been demonstrated, but Apatinib remains largely unexplored. In this study, whether Apatinib combined with homoharringtonine (HHT) kills AML cell lines and its possible mechanisms have been explored.

**Methods:**

AML cell lines were treated with Apatinib and HHT in different concentrations with control, Apatinib alone, HHT alone, and Apatinib combined with HHT. The changes of IC_50_ were measured by CCK8 assay, and apoptosis rate, cell cycle, and the mitochondrial membrane potential in each group were measured by flow cytometry. Finally, the possible cytotoxicity mechanism was analyzed by Western blotting.

**Results:**

Our results noted that Apatinib combined with HHT remarkably inhibited cell proliferation, reduced the capacity of colony-forming, and induced apoptosis and cell cycle arrest in AML cells. Mechanistically, Apatinib and HHT play a role as a suppressor in the expression of VEGFR-2 and the downstream signaling cascades, such as the PI3K, MAPK, and STAT3 pathways.

**Conclusion:**

Our preclinical data demonstrate that Apatinib combined with HHT exerts a better antileukemia effect than Apatinib alone by inhibiting the VEGFR-2 signaling pathway, suggesting the potential role of Apatinib and HHT in the treatment of AML. This study provides clinicians with innovative combination therapies and new therapeutic targets for the treatment of AML.

## 1. Introduction

Acute myeloid leukemia (AML) is derived from leukemia stem cells or progenitor cells. The American Cancer Society found that there are about 19, 940 new cases of AML per annum with 11,180 deaths [1]. Although it has significantly improved the molecular biology of this disease and over the last forty years treatment has changed, the outcome remains poor for most patients [2]. Cytogenetic abnormalities are closely associated with clinical features and therapeutic responses in AML [3]. Therefore, there is a crying need for novel AML therapies, ranging from drugs targeting specific vascular endothelial growth factors (VEGF), and oncogenic proteins to immunotherapies. In the last few years, a large number of studies have shown that angiogenesis is involved not only in leukemogenesis but also in leukemia progression [4]. An increased angiogenesis in the bone marrow niche relates to acute myeloid leukemia progression and resistance to treatment [5–9]. In consideration of the important role of angiogenic activity in different kinds of tumor-like hematological malignancies, targeting vasculogenesis signaling has gained more and more attention as a new therapy to avoid cancer metastasis and resistance. Therefore, antiangiogenic drugs or VEGFR inhibitors may provide a novel innovative approach for AML treatments [10–12].

Apatinib (also known as YN968D1), a small-molecule receptor tyrosine kinase inhibitor, can target VEGFR-2 selectively [13], which has been approved in China as a subsequent treatment for advanced gastric cancer [14]. Moreover, it has also been tested in phase II/III clinical trials of other cancers, including non-small-cell lung cancer and breast cancer [15]. In this case, we are intrigued to explore whether Apatinib can be used for AML treatment and its relevant mechanisms. Homoharringtonine (HHT) (also known as omacetaxine mepesuccinate), a classical antileukemia drug, has been applied for about forty years in China. HHT has a variety of antitumor effects, including AML [16] and CML [17]. The researchers found that HHT is a protein synthesis inhibitor affecting leukemic cells, which potentiates the therapeutic efficacy of anthracycline and cytarabine [18]. In China, HHT has been widely used in the treatment of AML for more than 30 years because of its good curative effect and low treatment cost [19]. Although HHT has been used for the treatment of a variety of tumors, the specific targets are still unknown [18, 20]. In this paper, we studied whether HHT would strengthen the antileukemic effect when combined with Apatinib. Besides, we also studied the underlying mechanisms of the cooperative effect of both two drugs.

## 2. Materials and Methods of Drugs

Apatinib (Houston, TX, USA). HHT (Zhejiang Minsheng Pharmaceutical, Zhejiang, China) was dissolved in dimethyl sulfoxide (DMSO) at 1 mg/mL and stored at −20°C. HHT was diluted with a culture medium in subsequent experiments.

### 2.1. Cell Culture

MV4-11, MOLM-13, OCI-AML2, and OCI-AML3 were purchased from the American Type Culture Collection (ATCC, Manassas, VA, USA). MV4-11 and MOLM-13 expressed MLL fusion oncoprotein with FLT3-ITD. OCI-AML2 and OCI-AML3 expressed mutant NPM1c+ THP1 without FLT3-ITD or NPM1c + mutant, all cells were cultured in RPMI 1640 with 20% fetal bovine serum (Gibco).

### 2.2. Cell Viability Assay

AML cell lines (2 × 10^4^ cells/well) were plated in 96-well plates and then treated with different concentrations of Apatinib and HHT for 24 h and 48 h. The cytotoxic effect was determined by cell counting kit-8 (CCK8; Dojindo, Japan) assay. IC_50_ (half-maximal inhibitory concentration) values were determined using a microplate reader (BIO-TEK EPOCH, USA).

### 2.3. Apoptosis Assay

To assess apoptosis, MV4-11, MOLM-13, OCI-AML2, and OCI-AML3 cells were cultured and presented with different doses of Apatinib or HHT alone or in combination for 24 h or 48 h then double-labeled with Annexin V/DAPI (eBioscience). However, the same concentration did not work in non-FLT3-ITD mutations in AML cell lines, for example, THP1. The stained cells were analyzed with a NovoCyte flow cytometer (ACEA Biosciences, Inc.) with NovoExpress software. Annexin V-positive cells were defined as apoptotic cells.

### 2.4. In Vitro Clonogenicity Assay

MV4-11, MOLM-13, OCI-AML2, and OCI-AML3 cells (2 × 10^5^/well) were used to test colony-forming abilities. AML cells were seeded in 24-well plates and then, treated with 20 µM Apatinib or 16 nM HHT alone or both molecules. After 24 h, cells were washed and then cultured in a complete methylcellulose medium at a cell density of 500 cells/well in 3.5 cm dishes for 10–14 days. The percentage of CFU was determined by counting colonies (≥50 cells). Data were presented as the mean ± SD of three independent experiments.

### 2.5. Cell Cycle Analysis

AML cells were treated for 24 h, then cells were fixed overnight with 75% ethanol, washed with PBS three times, and then incubated in buffer containing 50 *μ*g/mL PI and 100 *μ*g/mL RNase A for 30 min at room temperature. Cells were resuspended with DAPI saline solution and subjected to flow cytometry (NovoCyte™).

### 2.6. Analysis of the Mitochondrial Membrane Potential

AML cells (2 × 10^5^/ml) were plated in 24-well plates treated with various concentrations of Apatinib and HHT. After 24 h, cells were stained with 2 *μ*M Rhodamine 123 (Beyotime) for 30 min. Then, after washing, mitochondrial membrane potential (MMP) was presented by flow cytometry (FACS Fortessa).

### 2.7. Western Blot Analysis

Cell protein was extracted with RIPA protein lysis buffer (Beyotime). MOLM-13 cells (2 × 10^5^/mL) were cultured with 20 *μ*M Apatinib or 16 nM HHT alone or combined for 24 h and 48 h. Related antibodies included *β*-actin, VEGFR-2, p-VEGFR-2, PI3K, Akt, CyclinA2, CyclinD1, P21, and BCL-2 (rabbit, 1 : 1000, Cell Signaling Technology). Blots were tested by the addition of a horseradish peroxidase (HRP)-conjugated secondary antibody. Signals were visualized using the ECL Western blotting detection kit (Gene-Flow, Staffordshire, UK).

### 2.8. Statistical Analysis

We used GraphPad Prism software v7.0 to analyze the data. All experiments performed at least three independent experiments. Multigroup comparisons were using a one-way test of variance (ANOVA). Statistical analyses were presented using SPSS 20.0 software (La Jolla, CA).

## 3. Results

### 3.1. The Cytotoxic Effect of Apatinib and HHT on FLT3-ITD Mutations AML Cell Lines

**Figure 1:**
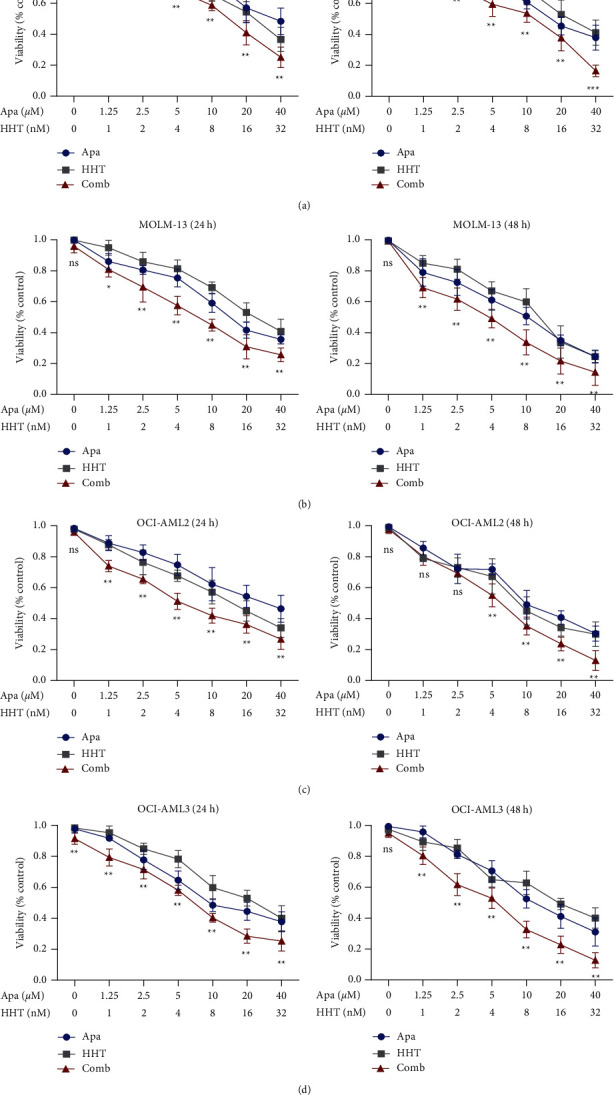
Cell viability after treatment with various doses of Apatinib or HHT alone or in combination for 24 h or 48 h. (a–d) The percent viability is normalized to the percent viability of the DMSO-treated control. Values are expressed as the mean ± SD of three independent experiments. The half-maximal inhibitory concentration (IC_50_) values of Apatinib and HHT in a time-dependent manner on FLT3-ITD mutations AML cells. ^*∗*^*p* < 0.05; ^*∗∗*^*p* < 0.01; ^*∗∗∗*^*p* < 0.001.

We used the CCK8 assay to test the activity of Apatinib and HHT alone and in combination to verify the synergistic effect in inhibition of FLT3-ITD mutations AML cell viability. The concentrations of Apatinib and HHT are shown in Figures 1(a)–1(d). We found that AML cell lines treated with both Apatinib and HHT showed a much better inhibitory effect, especially in the FLT3-ITD group than those treated with each reagent alone in a time-dependent manner. However, Apatinib and HHT displayed no effect on the THP1 cell line without FLT3-ITD mutations in cell proliferation (Supplemental Figure 1). The IC_50_ values for Apatinib and HHT were calculated using GraphPad Prism software v7.0 in AML cells, respectively (Table 1). Together, these results indicate that Apatinib synergistically interacts with HHT to reduce the viability of AML cells.

### 3.2. Combination Treatment with Apatinib and HHT Plays a Synergistic and Lethal Role in AML Cell Lines Especially in FLT3-ITD Mutations in AML Cells

**Figure 2:**
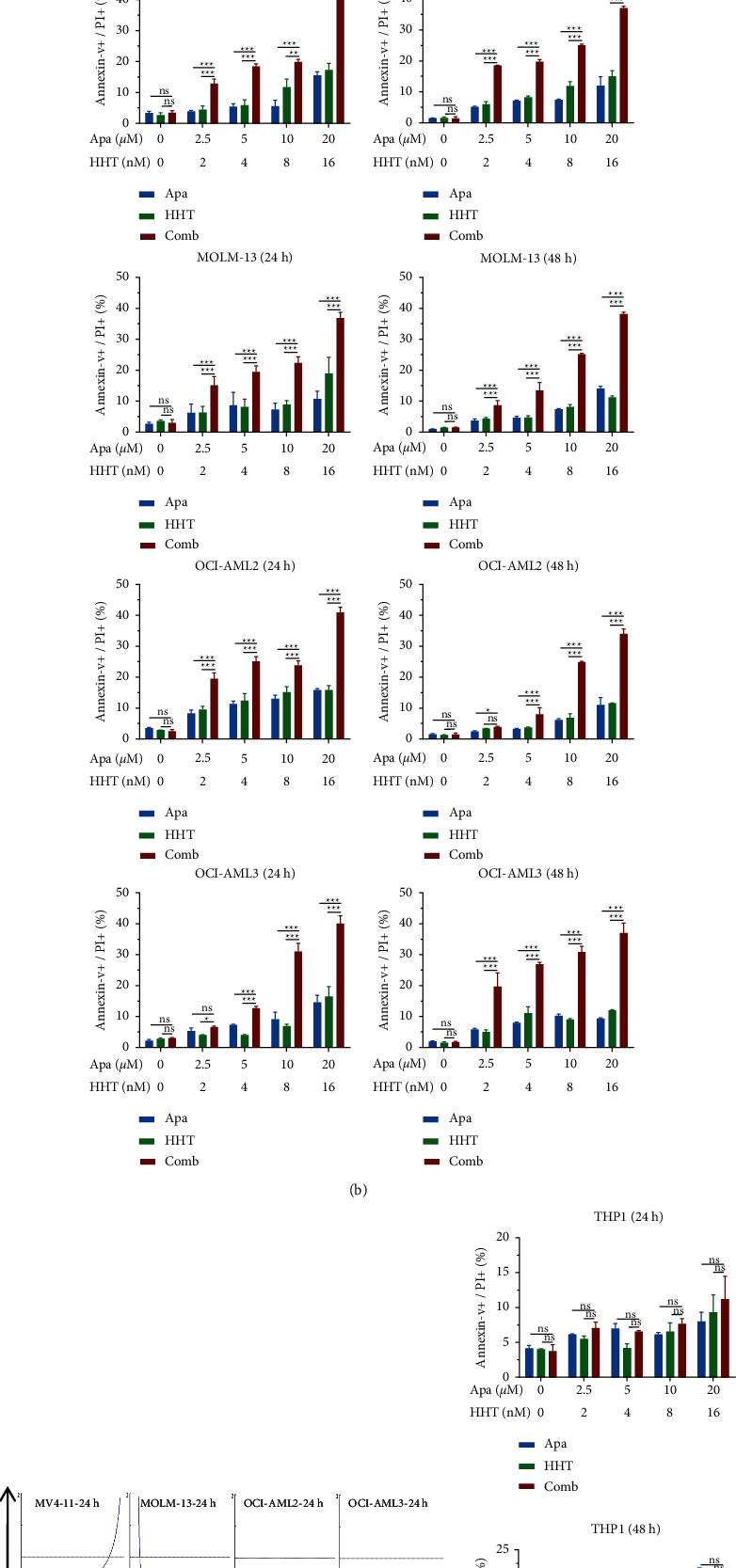
Coexposure to Apatinib and HHT induces apoptosis of AML cells. (a) The percentage of apoptotic cells was examined with a NovoCyte flow cytometer. (b) Cells were treated with the indicated concentrations of Apatinib ± HHT for 24 and 48 h after which the percentage of Annexin-V + apoptotic cells was determined by flow cytometry after annexin V and DAPI double staining. (c) Combination index (CI) values were calculated according to the median effect method of Chou and Talala. (d) The combination of Apatinib and HHT displayed no effect on the THP1 cell line without FLT3-ITD mutations. ^*∗*^*p* < 0.05; ^*∗∗*^*p* < 0.01; ^*∗∗∗*^*p* < 0.001.

To explore the synergistic effect of Apatinib and HHT on FLT3-ITD AML cells. Annexin V/DAPI staining was then performed to examine whether Apatinib would enhance HHT to induce apoptosis in FLT3-ITD AML cells. MV4-11, MOLM-13, OCI-AML2, and OCI-AML3 cells were exposed to the defined concentrations of Apatinib and HHT for 24 h or 48 h. As shown in Figures 2(a) and 2(b), Apatinib or HHT alone was unable to induce apoptosis, while the combination could significantly increase apoptosis in all FLT3-ITD AML cell lines. Combination index (CI) values were calculated using the Chou and Talala software (Figure 2(c)) and (Table 2). A CI value of less than 1.0 means a synergistic effect. In contrast, the combination of Apatinib and HHT displayed no effect on the THP1 cell line without FLT3-ITD mutations (Figure 2(d)). These findings suggested that the combination of Apatinib and HHT might be a hopeful therapy for AML with FLT3-ITD mutation cells.

### 3.3. The Synergistic Effects of Apatinib Combined with HHT on the Formation of Colonies

**Figure 3:**
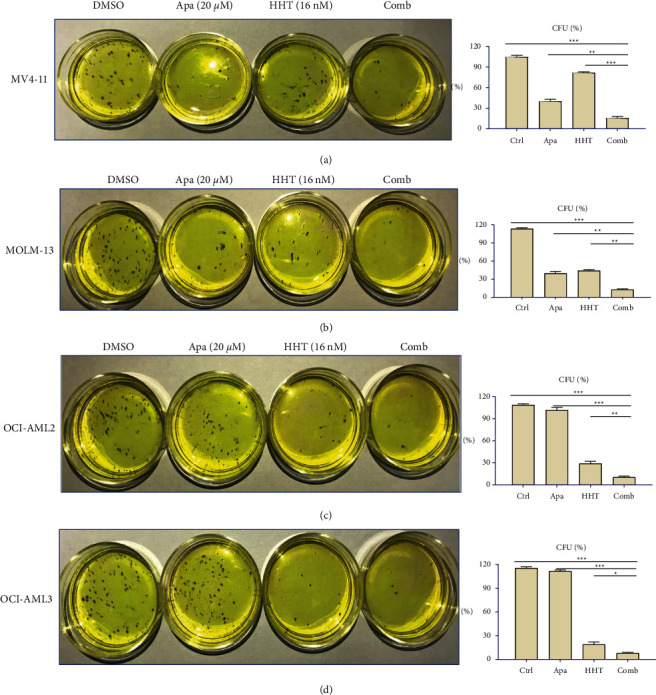
Orming abilities. (a–d) AML cells were seeded in 24-well plates and then treated with 20 *μ*M Apatinib or 16 nM HHT alone or both molecules. After 24 h, cells were washed and then cultured in a complete methylcellulose medium at a cell density of 500 cells/well in 3.5 cm dishes for 10–14 days. The percentage of CFU was determined by counting colonies (≥50 cells). Data are presented as the mean ± SD of three independent experiments. ^*∗*^*p* < 0.05; ^*∗∗*^*p* < 0.01; ^*∗∗∗*^*p* < 0.001.

Next, we studied the effects of Apatinib, HHT, or the combination of these two drugs on the cell colony formation of FLT3-ITD mutation cells. MV4-11, MOLM-13, OCI-AML2, and OCI-AML3 cells were treated with different concentrations of Apatinib or HHT alone or in combination for 48 h. Neither Apatinib (20 *μ*M) nor HHT (16 nM) alone diminished the colony formation abilities of FLT3-ITD mutations in AML cells. However, when Apatinib was combined with HHT, the colony-forming units significantly decreased (*p* < 0.001 vs. control, Apatinib alone, or HHT alone) (Figures 3(a)–3(d)).

### 3.4. Apatinib and HHT Induce Cell Cycle Arrest in FLT3-ITD Mutations AML Cells

**Figure 4:**
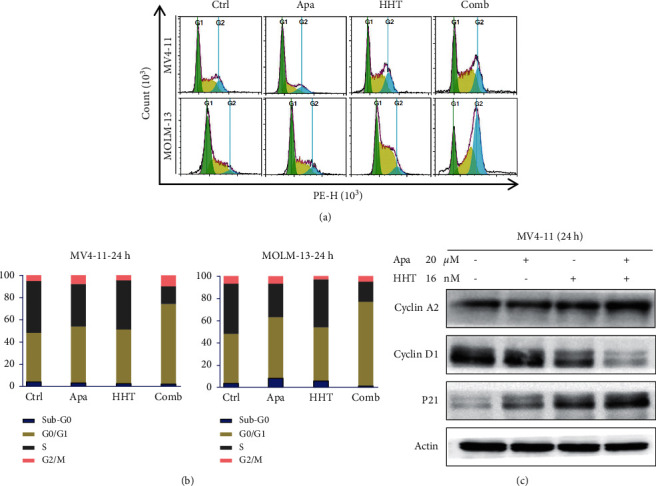
In MV4-11 and MOLM-13 cells, G0/G1 phase arrest and S phase decrease were observed after treatment with Apatinib and HHT. (a, b) After drug treatment for 24 h, cell cycle distribution was analyzed using flow cytometry (^*∗*^*p* < 0.05, one-way ANOVA, combination treatments versus control, and single treatments). (c) Western blot analysis showed an increase in cell cycle regulator CyclinA2, CyclinD1, and P21 in cells treated with Apatinib and HHT compared to a single agent. actin served as a loading control.

Cell cycle assays were carried out to investigate whether Apatinib combined with HHT affects the cell cycle capacity in FLT3-ITD mutation cells. MV4-11 and MOLM-13 cells were treated with different concentrations of Apatinib alone or combined with HHT for 24 hours. As shown in Figures 4(a) and 4(b), cells treated with Apatinib at 20 *μ*M or HHT at 16 nM, these concentrations did not affect G0/G1 or S phase cells notably. However, Apatinib and HHT exerted a prominent G0/G1 phase arrest and the S phase decreased. Then, we used Western blots to explore the cycle-relevant proteins, such as cyclin A2, cyclin D1, and P21, and the results were consistent with the former (Figure 4(c)). Consistent with the cell cycle assays, cell cycle-relevant proteins of Apatinib plus HHT induced a reduction of cyclin D1 and a rising of P21. Also, we found cyclin A2 was increased, which was a relation to the fewer cycling cells in the S phase.

### 3.5. Apatinib Combined with HHT Dose-Dependent Manner Reduces the Mitochondrial Membrane Potential

**Figure 5:**
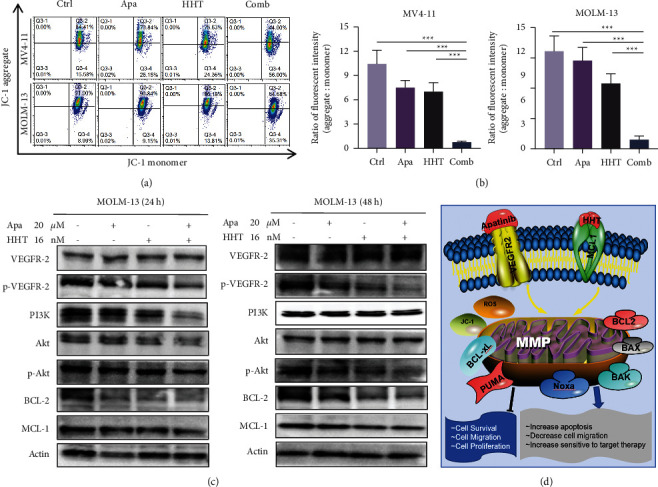
In AML cell lines, Apatinib and HHT decreased mitochondrial membrane potential and altered p-VEGFR-2, PI3K/Akt pathway associated proteins. (a, b) MV4-11 and MOLM-13 cells with loss of mitochondrial membrane potential. (c) MOLM-13 cells were exposed to the indicated concentrations of Apatinib ± HHT for 24 h and 48 h after which western blot analysis was shown to monitor the expression of p-VEGFR-2, PI3K/Akt pathway associated proteins, anti-apoptotic proteins BCL-2 and MCL-1. (d) pharmacologic targeting VEGFR-2 and MCL-1 induces mitochondrial dysfunction, decreased intracellular MMP levels, and apoptosis in AML.

We used the JC-1 probe to test the mitochondrial membrane potential (MMP, ΔΨm) to validate the joint effects of Apatinib and HHT. As we expected, compared with each reagent alone, co-treatment with Apatinib and HHT remarkably reduced the MMP in MV4-11 and MOLM-13 cells after 24 h treatment (Figures 5(a) and 5(b)). Apatinib combined with HHT downregulates the VEGFR-2 and its downstream signaling pathways in AML cells. As we mentioned above, Apatinib was a selective target to the VEGFR-2 pathway. What surprises us was that when Apatinib was combined with HHT the VEGFR-2 expression was affected. Apatinib and HHT treatment markedly inhibited the downstream signals, PI3K and p-Akt, as well as antiapoptotic proteins like BCL-2 and MCL-1 (Figure 5(c)). We can summarize the mechanism through a brief schematic mechanism as follows: on FLT3-ITD mutations AML cells Apatinib combined with HHT induced cell apoptosis by decreasing the mitochondrial membrane potential, inhibiting cell cycle, and regulating vascular endothelial growth factor as well as its downstream signaling pathways (Figure 5(d)).

## 4. Discussion

The evidence is overwhelming that the poor prognosis and higher disease relapse rate of AML accompanied by FLT3-ITD mutations make FLT3-ITD a perfect therapeutic target in individualized treatment [21–23]. Through our experimental results, we found that on FLT3-ITD (+) AML cell lines, compared to the monotherapy group, Apatinib and HHT could significantly inhibit cell proliferation, promote cell apoptosis, and regulate Apatinib-relevant protein VEGFR-2. However, these results were not found on the FLT3-ITD (-) THP1 cell line. Hence, we proposed and proved for the first that the combination of Apatinib and HHT exerted a significant antileukemic action. Mechanistically, the combination of Apatinib and HHT synergistically decreases phosphorylated forms of VEGFR-2 protein and its downstream PI3K, BCL-2, Akt, and MCL-1, resulting in cell arresting at G1 and apoptosis. As we know, approximately 30% of AML with normal karyotype will have FLT3 (FMS-like tyrosine kinase 3) gene with mutations of internal tandem duplications (ITD) in the juxtamembrane domain [24]. Meanwhile, AML with FLT3-ITD mutations is concerned with poor overall survival (OS) and decreased disease-free survival (DFS) [25]. Although FLT3-mutant AML patients can be treated with FLT3 tyrosine kinase inhibitors (TKI), the relapse and rapid drug resistance limit its use [26]. Lately, several studies have found that HHT exerted a sensitive cytotoxic function on FLT3-ITD (+) AML cells [27].

The key role of angiogenesis is the process of forming blood vessels in the growth and maintenance of solid tumors. In the last few decades, a large number of studies show the involvement of angiogenesis in leukemogenesis as well as leukemia progression [28]. An increase in angiogenesis in the bone marrow niche is related to both acute lymphoblastic leukemia and acute myeloid leukemia [5–9]. Therefore, targeting angiogenesis with antiangiogenic agents or VEGFR inhibitors is likely to be a new method for AML treatment. Apatinib is a novel small-molecule tyrosine kinase inhibitor, which inhibits the phosphorylation of vascular endothelial growth factor receptors selectively, with a binding affinity ten times that of sorafenib. Besides, compared with Apatinib sorafenib with only one-tenth of the anti-VEGFR-2 efficacy, it is insufficient for antitumor angiogenesis [10, 14, 29]. Its antitumor activity in all kinds of tumors has been proved in many studies [20, 30, 31]. Results of those clinical trials indicated the antitumor role of Apatinib across a large scale of advanced cancers, but the specific function of tumor angiogenesis in AML pathogenesis remains unknown. Furthermore, our research has found that Apatinib combined with a variety of antitumor drugs can improve the curative effect [32, 33]. It was interesting to note that accompanied FLT3-ITD mutant AML cells presented a significant synergistic effect after being treated with Apatinib and HHT. Nevertheless, it makes no difference to the AML cell line, which was without FLT3-ITD mutant (Figure 2(d)). It has been well reported that the important role of PI3K signaling in the progression of all kinds of tumors, including leukemia [34, 35]. The evasion of apoptosis was an important characteristic of cancer, which was caused by the activation of antiapoptotic molecules of the BCL-2 protein family [36–38]. Aberrant activation of BCL-2 members such as BCL-2 and MCL-1 were related to antiapoptosis and drug resistance in FLT3-ITD mutant AML [39–41].

VEGF is considered a target in leukemia treatment and a variety of strategies have been applied to downregulate or inhibit the VEGF signaling pathway. The new strategy which inhibits the VEGF signaling pathway could be able to block the autocrine VEGF pathway in AML cells or the typical vessel development by the vascular endothelial cells [42]. We and other researchers found that Apatinib-induced cytotoxicity was related to inhibition of the VEGFR-2 and PI3K/Akt pathways, and induction of mitochondrial membrane protein (MMP)-mediated apoptosis. To our surprise, Apatinib and HHT could significantly enhance this phenomenon. As everyone knows that FLT3-ITD mutations result in missing the autoinhibitory function for FLT3 kinase, which next leads to activation of its downstream signaling pathways, such as PI3K/Akt and JAK/STAT5 [43, 44], our results indicated that Apatinib combined with HHT synergistically suppressed the growth and induced apoptosis of FLT3-ITD mutations AML cells by synergistically downregulating the expression of phosphorylated forms of VEGFR-2 and PI3K/Akt signaling pathways as well as affecting the expression level of cell cycle regulatory protein, upregulating the expressions of cyclin A2 and P21 and downregulating the expression of cyclin D1 in FLT3- ITD (+) AML cell lines. Nevertheless, mechanisms of combined application of Apatinib and HHT in regulating P21, cyclin A2, and cyclin D1 proteins remain unclear and need further study.

## 5. Conclusions

All in all, our research first reveals the synergistic antileukemic effect between Apatinib and HHT on FLT3-ITD mutant AML cells, likely through inhibiting VEGFR-2-mediated signaling pathways, and suggests potential benefits and clinical application of Apatinib combined with HHT in the treatment of AML patients.

## Figures and Tables

**Table 1:** IC_50_ values of Apatinib and HHT as single agent in AML cells.

AML cell lines	IC_50_ at 24 h		IC_50_ at 48 h	
	Apa (*μ*M)	HHT (nM)	Apa (*μ*M)	HHT (nM)
MV4-11	6.76 ± 1.14	15.2 ± 1.80	4.67 ± 0.81	6.05 ± 1.31
MOLM-13	4.11 ± 0.81	12.2 ± 2.83	2.25 ± 1.22	5.30 ± 1.04
OCI-AML2	101.1 ± 11	16.2 ± 0.89	31.2 ± 15.8	7.69 ± 1.28
OCI-AML3	4.97 ± 1.30	15.93 ± 1.17	3.23 ± 0.56	5.51 ± 0.91

**Table 2:** The effect of synergistic inhibition in AML cell lines.

MV4-11					
Concentration		24 h		48 h	
Apa	HHT	Fa	CI	Fa	CI

2.5	2	0.14	0.1927	0.36	0.1244
5	4	0.18	0.2579	0.38	0.2191
10	8	0.19	0.4723	0.50	0.2153
20	16	0.39	0.2584	0.72	0.1136

MOLM-13					
Concentration		24 h		48 h	
Apa	HHT	Fa	CI	Fa	CI

2.5	2	0.12	0.5103	0.14	0.5988
5	4	0.17	0.3835	0.22	0.5731
10	8	0.20	0.5256	0.49	0.2373
20	16	0.38	0.2088	0.75	0.1181

OCI-AML2					
Concentration		24 h		48 h	
Apa	HHT	Fa	CI	Fa	CI

2.5	2	0.18	0.1715	0.08	0.9547
5	4	0.24	0.128	0.20	0.4447
10	8	0.25	0.2212	0.50	0.1198
20	16	0.39	0.0764	0.71	0.0660

OCI-AML3					
Concentration		24 h		48 h	
Apa	HHT	Fa	CI	Fa	CI

2.5	2	0.07	0.5814	0.28	0.080
5	4	0.14	0.3159	0.35	0.0563
10	8	0.28	0.1578	0.42	0.0435
20	16	0.37	0.1682	0.57	0.0126

Fa: fraction affected.

## Data Availability

The datasets used and/or analyzed during the current study are available from the corresponding author on reasonable request.

## Abbreviations

AML: Acute myeloid leukemia
HHT: Homoharringtonine
VEGFR-2: Vascular endothelial growth factor receptor-2 (VEGFR-2)
CI: Combination index.

## Supplementary Materials

Supplementary Materials Supplemental Figure 1: Apatinib and HHT displayed no effect on the THP1 cell line without FLT3-ITD mutations in cell proliferation.
