# Supplementary material for: Synergistic Lethality Effects of Apatinib and Homoharringtonine in Acute Myeloid Leukemia
Source: J Oncol. 2022 Aug 30;2022:9005804. doi: 10.1155/2022/9005804 (PMC9448536; doi:10.1155/2022/9005804)
Supplement: Supplementary Materials — Supplemental Figure 1: Apatinib and HHT displayed no effect on the THP1 cell line without FLT3-ITD mutations in cell proliferation. [file 9005804.f1.docx]

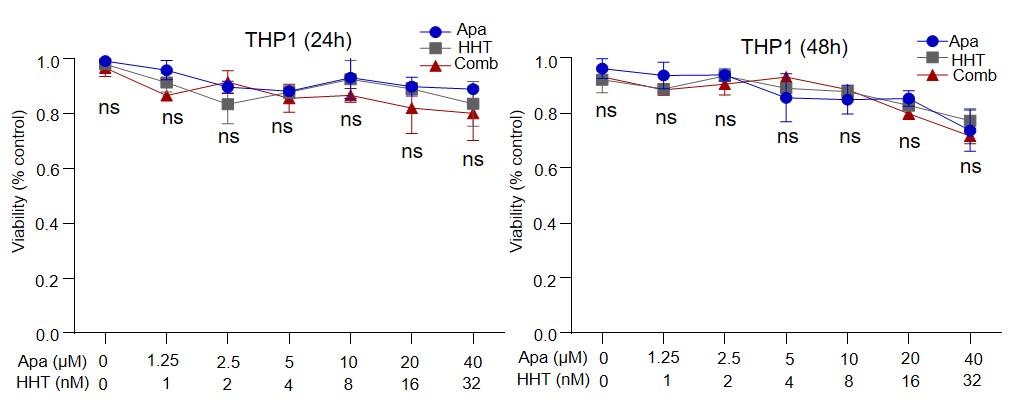


**Supplemental Figure 1** Apatinib and HHT displayed no effect on the THP1 cell line without FLT3-ITD mutations in cell proliferation.
